# Selective Wet-Etching of Polymer/Fullerene Blend Films for Surface- and Nanoscale Morphology-Controlled Organic Transistors and Sensitivity-Enhanced Gas Sensors

**DOI:** 10.3390/polym11101682

**Published:** 2019-10-15

**Authors:** Min Soo Park, Alem Araya Meresa, Chan-Min Kwon, Felix Sunjoo Kim

**Affiliations:** School of Chemical Engineering and Materials Science, Chung-Ang University, Seoul 06974, Korea; vvifro06@naver.com (M.S.P.); alemaraya12man@gmail.com (A.A.M.); chanminkwon91@gmail.com (C.-M.K.)

**Keywords:** polymer blend, selective etching process, morphology, organic thin-film transistor, chemical sensor

## Abstract

Surface and nanoscale morphology of thin poly(3-hexylthiophene) (P3HT) films are effectively controlled by blending the polymer with a soluble derivative of fullerene, and then selectively dissolving out the fullerene from the blend films. A combination of the polymer blending with fullerene and a use of diiodooctane (DIO) as a processing additive enhances the molecular ordering of P3HT through nanoscale phase separation, compared to the pristine P3HT. In organic thin-film transistors, such morphological changes in the blend induce a positive effect on the field-effect mobility, as the mobility is ~5–7 times higher than in the pristine P3HT. Simple dipping of the blend films in butyl acetate (BA) causes a selective dissolution of the small molecular component, resulting in a rough surface with nanoscale features of P3HT films. Chemical sensors utilizing these morphological features show an enhanced sensitivity in detection of gas-phase ammonia, water, and ethanol.

## 1. Introduction

Sensor technology has become of great interest for the wide-spread needs of monitoring various chemicals, physical signals, biological species, and environmental conditions [1,2,3,4]. Among diverse platforms of signal transduction, organic thin film transistors (OTFTs) are considered one of the most convenient sensing elements, because it directly converts the signal into a form of electricity, and one can chemically fine-tune the materials and devices for better sensitivity and selectivity [5,6,7,8,9,10]. In typical OTFT-based chemical sensors, the organic semiconductor non-covalently interacts with analytes in a form of charge transfer or dipolar interaction. As these physical and chemical interactions between the semiconductor and the analytes affect the charge transport properties, the electrical signals in the OTFTs can be easily modulated. OTFT-based active sensors can also simultaneously amplify the signals during transduction, making the devices more sensitive compared to other passive systems. OTFTs are especially promising for sensing gas-phase analytes such as volatile organic compounds (VOCs) because, unlike in a liquid-phase or solution, a solubility of the organic electro-active film is not an issue. As an active material for OTFT-based sensors, poly(3-hexylthiophene) (P3HT) has become a benchmarking and reference material in testing various engineering approaches of OTFT-based sensors, because the material is commercially available and widely investigated [11,12,13,14,15,16,17,18,19,20]. We note that, although P3HT has been used for many proof-of-concept works, various combinations of materials, device structures, and processing methods would bring high-performance sensor systems closer to real implementations, as there have been remarkable developments in the area of conjugated polymers and semiconductors [21,22,23].

There have been noticeable strategies to improve the performance of the OTFT-based sensors. The selectivity of OTFT-based sensors designated for detection of specific chemicals can be improved from a wide range of organic semiconductors, or by designing novel materials and tailoring the structures suitable for interaction with the target analyte [24,25,26,27,28,29,30]. Such materials include new organic and polymer semiconductors, specific receptors with complementary structures, and selective molecule-transporting layers. One can also externally modulate the morphology to have a better sensitivity [20,31,32,33,34,35,36,37]. Highly crystalline micro- and nanostructures of polymers have showed a higher sensitivity towards analytes [14,20]. For the sensitivity enhancement, one can aim for a high surface or interfacial area of the sensing element. Different processing techniques have been developed for the purpose of morphological control of organic and polymer semiconductors.

To achieve desired selectivity and high sensitivity to a specific analyte, new materials with complementary binding sites should be developed. However, designing materials for specific VOCs that are commonly observed in living, industrial, and agricultural conditions will be very challenging because of their simple and similar structures with common functional groups. A more practical approach to pin-point the specific analyte among VOCs is to obtain a molecular fingerprint of the compound using arrays of sensors [4,5,14]. Because various semiconductors can show different responses depending on the analytes, a series of available polymers can spread over the sensor matrix. Then the sensitivity becomes a more critical property to be enhanced for diverse materials.

In this work, we present a blend system of P3HT with phenyl-C61-butyric acid methyl ester (PCBM) and selective removal of the small-molecular component by a wet-etching process to control the surface and nanoscale thin-film morphology of the polymer semiconductor. Depending on the presence of the processing additive, the molecular ordering of polymer and the charge transport properties of the films are greatly influenced. Selective etching of PCBM resulted in a rough surface with nanoscale features. Because gas-phase chemical species can easily access the charge-transporting channel through the nanostructured surface, we investigated the sensitivity of the polymer films to commonly-observed analytes, such as ammonia, water, and ethanol. We quantified the sensing response by the change in the relative field-effect mobility and found that the morphology-controlled sensors showed superior sensitivity, with a larger decrease in the relative mobility upon analyte exposure.

## 2. Materials and Methods

### 2.1. Preparation of Polymer Thin Films

Regioregular poly(3-hexylthiophene-2,5-diyl) (P3HT; 50–70 kg/mol and 91–94% regioregularity) and phenyl-C61-butyric acid methyl ester (PCBM) were purchased from Rieke Metals, Inc. (Lincoln, NB, USA) and Nano-C (Westwood, MA, USA), respectively. Chloroform, *n*-butyl acetate (BA), trichloroethylene, octyltrichlorosilane (OTS), and 1,8-diiodooctane (DIO) were purchased from Sigma-Aldrich (St. Louis, MO, USA). A piece of clean wafer consisting of highly doped Si with a thermally grown 200 nm SiO_2_ or slide glass was used as a substrate. The substrate was hydrophobically modified by spin coating of an OTS solution in trichloroethylene (5 mM) at 2–3 krpm for 20 s, followed by annealing at 100 °C for 10 min in air. Solutions of P3HT and P3HT:PCBM blend (1:1 by weight) were prepared at 10 mg/mL in chloroform. To control the thin-film morphology of blends, 2.5 vol% of DIO was added to the P3HT:PCBM solution. Thin films of active polymer and blends were deposited onto the substrate by spin coating of the solution at 2 krpm for 60 s under nitrogen environment, followed by annealing at 120 °C for 30 min. For selective etching of PCBM, the thin films were immersed in a bath of BA for 15 s, followed by spin-drying at 7 krpm for 40 s. For device fabrication, source and drain electrodes were defined by thermal evaporation of gold (~50 nm) through a shadow mask. The channel width was 1 mm and the length was either 0.05 mm or 0.1 mm. The contact pads of electrodes were manually formed with pieces of indium to ensure the proper electrical connection. The polymer and blend films are exposed in ambient air and conventional laboratory conditions without high-intensity light during transfer between glove box, thermal evaporator, and characterization chamber.

### 2.2. Characterization

UV-Vis spectra were obtained by using a spectrophotometer (V-770, JASCO, Inc., Easton, MD, USA). Surface morphology and nanostructures of thin films were imaged by using an atomic force microscope (AFM, XE-100, Park Systems, Suwon, Korea) and an X-ray diffractometer (XRD, D8-Advance, Bruker, Billerica, MA, USA). The XRD data was analyzed by fitting with pseudo-Voigt functions. X-ray photoelectron spectroscopy (XPS) was performed by using an Axis Supra spectrometer (Kratos Analytical, Manchester, UK). The transistor and sensor devices were tested by using a custom-made probe station and a semiconductor parameter analyzer (HP4156A). The field-effect mobility (*μ*) and the threshold voltage (*V*_T_) of the devices were calculated from the curves of the saturation regime. Analyte exposure was conducted at room temperature in a hand-made chamber (16 L) with manual valves for gas inlet, outlet, and vacuum lines. The analyte, such as vaporized 1-M ammonium hydroxide, deionized water, or ethanol, was introduced into the chamber with nitrogen as a carrier gas to control the gas-phase concentration. The analyte concentration in a chamber was calculated with an assumption of the ideal gas.

## 3. Results

**Figure 1:**
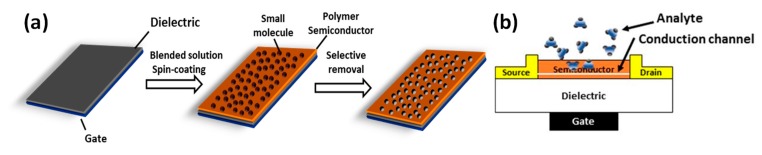
(**a**) Illustrative diagram presenting fabrication of morphology-controlled polymer films by selective dissolution of a small molecular component. (**b**) Schematic of chemical sensors based on the organic thin-film transistor.

Our approach to control the surface morphology of thin polymer films involves two step processing, deposition of polymer/small molecule blend and selective etching of the small-molecular component (Figure 1). We adopted blends based on a polymer (i.e., P3HT) and a small molecule (i.e., PCBM), because the P3HT:PCBM system has been widely investigated in the areas of organic electronics and solar cells, and various processing conditions and additives have been utilized to control their morphology and electronic properties [38,39,40,41,42,43,44]. We first spin-coated the polymer and blends dissolved in chloroform, resulting in the polymer films with the thickness ranging ~50–110 nm. Since a processing additive such as DIO is known to affect the morphology of polymer and polymer:PCBM blends, we prepared the blend both with and without DIO [42,43,44,45,46,47]. Our hypothesis was that, with DIO known to enhance the phase separation of the P3HT:PCBM mixtures and form PCBM agglomerates in polymer solar cells, the selective solvent can easily access the PCBM clusters and remove them from the polymer matrix [40]. Then, the prepared films were subjected to immersion in BA. This procedure selectively etches the PCBM component out of the active semiconductor layer, thereby leaving a rough surface of thin polymer films. With the removal of PCBM component from the polymer matrix, the film thickness decreased from 105.0 (±7.3) nm to 73.8 (±8.9) nm for the P3HT:PCBM, and from 110.8 (±2.1) nm to 71.3 (±6.3) nm for the blend with DIO. As depicted in Figure 1, the wet-etching process can result in an efficient and effective access to the chemical analytes.

**Figure 2:**
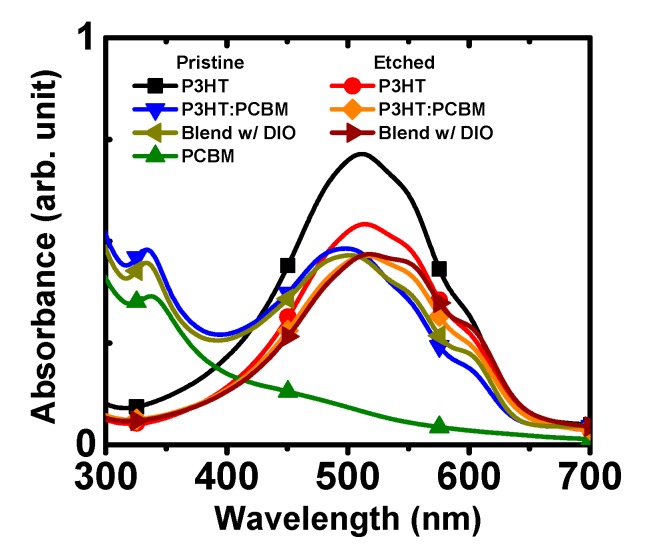
UV-Vis absorption of thin films of polymer and polymer blends under various conditions.

To ensure the selective removal of PCBM, UV-VIS absorption spectra were obtained from the spin-coated thin films of polymer and P3HT:PCBM blends, both before and after wet etching (Figure 2). P3HT and PCBM have characteristic absorption bands at ~500 nm and ~330 nm, respectively. The complementary absorption profiles of P3HT and PCBM allowed us to detect the main components in the blend films depending on the processing conditions. The absorption of P3HT:PCBM blends shows two peaks, and can be described as a simple superposition of the profiles of pristine P3HT and PCBM. However, when the blend sample was immersed in BA and then dried, the absorption peak corresponding to PCBM disappeared, leaving the polymer in the film. This change suggests that a majority of PCBM is selectively removed during the dipping process in BA, although there may be a small amount of PCBM remained. Comparing the blend films processed without and with DIO, the addition of a processing additive resulted in larger intensities of shoulders at 550 nm and 600 nm, relative the corresponding peak at 500 nm. These more prominent shoulders in the P3HT signal with the addition of DIO suggest a higher degree of interaction between P3HT molecules [47].

**Figure 3:**
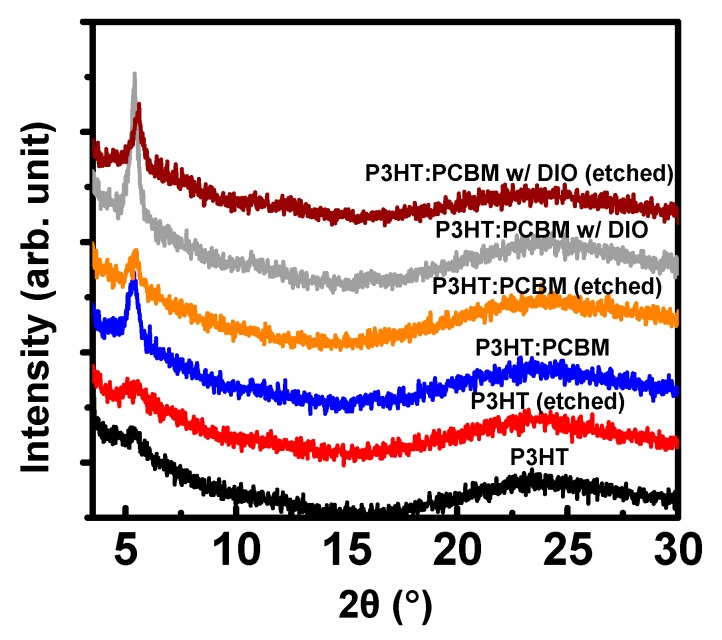
X-ray diffractometer (XRD) of thin films of the P3HT and P3HT:PCBM blends under various conditions.

We then investigated the X-ray diffraction of the polymer and blend films before and after etching (Figure 3). In a large angle of XRD, there is no meaningful features observed, and the signals are almost identical regardless of the processing conditions. There is a small diffraction peak at ~5°, corresponding ~1.6 nm of the *d*-spacing, in the pristine P3HT and the processed films under the given conditions. The BA treatment on the pristine P3HT films has little effect on the diffraction patterns. The same peak was also observed in P3HT:PCBM films. However, the incorporation of PCBM enhanced the intensity of the P3HT peak. The blend films with DIO have an even higher intensity of the peak. This observation could be related to the PCBM-assisted promotion of molecular arrangement of P3HT. After dipping of the films, however, both intensity and *d*-spacing become smaller compared to the pristine samples. The decrease in the diffraction intensity might have originated from partial collapse of the structures during the etching process. The effects of DIO are more prominent when we calculate the mean coherent length of the crystalline domains using the Scherrer equation after pseudo-Voigt curve fitting on the XRD data. The coherent lengths of the P3HT:PCBM films without and with DIO were 13.9 nm and 19.7 nm, respectively. The length slightly decreased to 16.7 nm after the PCBM etching, suggesting that the structures were partially influenced during the processing.

**Figure 4:**
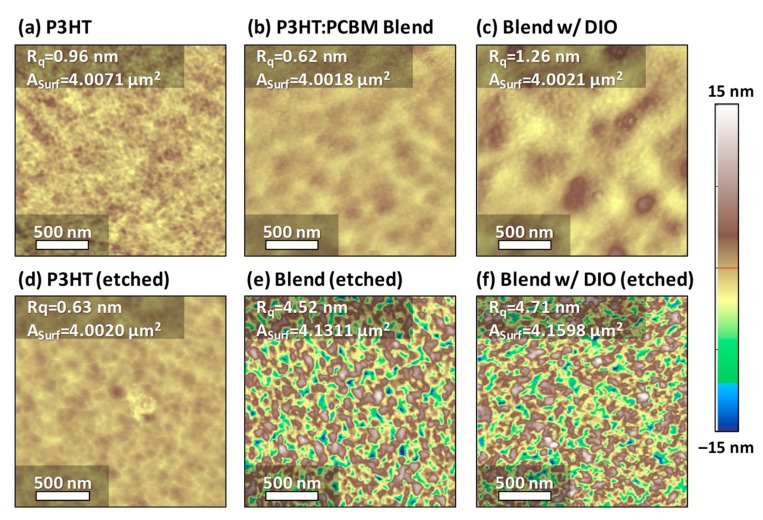
AFM topography images: (**a**–**c**) Pristine thin films of (**a**) P3HT, (**b**) P3HT:PCBM blend, and (**c**) P3HT:PCBM blend with 2.5 vol% 1,8-diiodooctane (DIO) as an additive. (**d**–**f**) Polymer and blend thin films after wet-etching by butyl acetate (BA). Note that the height difference is larger in (**e**) and (**f**). Polymer and blend films were cast from a solution in chloroform. The images share an identical scale bars.

Evidence of selective removal of small molecules from the blends by BA can be also found in the surface structures of the films, as shown in the AFM images (Figure 4). The root-mean-square roughness (R_q_) of the pristine P3HT film was 0.96 nm. The P3HT:PCBM blend has a slightly lower roughness of 0.62 nm, due to the PCBM as a filler. If the solidification of the P3HT:PCBM film was kinetically delayed through the high-boiling-point DIO, enhanced phase separation resulted in a higher roughness of 1.26 nm. These films of P3HT, P3HT:PCBM, and P3HT:PCBM with DIO showed dramatic changes in the roughness after BA dipping. The roughness of the P3HT film decreased from 0.96 nm to 0.63 nm when wet-etched with BA. This decrease suggests that, although BA dipping partially affects the surface roughness, the degree of decrease is not significant because BA is a poor solvent for P3HT. On the other hand, the roughness of the P3HT:PCBM thin films greatly increased from 0.62 nm to 4.52 nm after the etching process. As shown in the absorption (Figure 2), we can expect that PCBM is selectively etched out during the process, resulting in a rough surface of P3HT remained. Similarly, the roughness of the blends with an addition of DIO increased from 1.26 nm to 4.71 nm. It should be noted that, although large pores in the P3HT films may collapse during the PCBM-etching processes, the films can still maintain the nanostructures, as seen in the AFM images.

In order to further quantify the changes in the film surface depending on the processing conditions, we also calculated the surface area (A_Surf_) from the AFM images of 4 μm^2^, by using the AFM analysis software. In the pristine P3HT and P3HT:PCBM blends without and with DIO, the surface area values (4.00–4.01 μm^2^) are almost identical to the projected area (4 μm^2^). There are no meaningful changes in the area when the pristine P3HT was washed with BA. However, the PCBM etching on the blends resulted in a large increase in the surface area to 4.13 μm^2^ without DIO and 4.16 μm^2^ with DIO. In short, removal of PCBM by the etching process has a great influence on the surface morphology of the thin polymer films.

**Figure 5:**
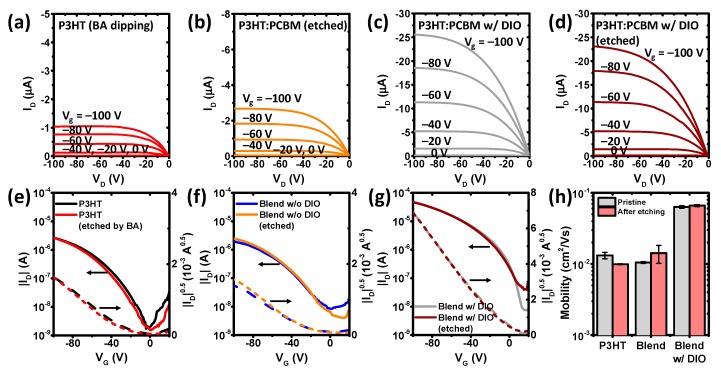
Comparison of organic thin film transistor (OTFT) characteristics of P3HT and P3HT:PCBM blends, with and without wet-etching process. (**a**–**d**) Output curves of (**a**) P3HT after BA dipping, (**b**) P3HT:PCBM after etching, (**c**) P3HT:PCBM with DIO without etching, and (**d**) P3HT:PCBM with DIO after etching. (**e**–**g**) Transfer curves of (**e**) P3HT, (**f**) P3HT:PCBM, and (**g**) P3HT:PCBM processed with DIO. (**h**) Field-effect hole mobility of the devices. Device testing conditions: *W*/*L* = 10 and *V*_D_ = −100 V.

We then fabricated and tested organic thin-film transistors based on the surface-controlled thin films as an active layer. As shown in Figure 5, the OTFTs based on the polymer and blends showed typical p-type characteristics with a current modulation larger than 10^3^. The performance of OTFTs was influenced by the etching processing. We initially observed the hole mobility of 0.0132 (±0.0014) cm^2^ V^−1^ s^−1^ in the pristine P3HT films. This value is within a reasonable range for P3HT, because the molecular weight, regioregularity, purity, device structure, and deposition condition largely affect the P3HT mobility. The P3HT:PCBM blends have the mobilities of 0.0105 (±0.0003) cm^2^ V^−1^ s^−1^ without DIO, and 0.0633 (±0.0027) cm^2^ V^−1^ s^−1^ with DIO. The hole mobility is much higher for P3HT:PCBM with DIO. This increase in mobility can be explained as a result of higher degree of molecular organization in solid state, as observed in the UV/Vis and XRD. We note that there is no evidence of electron transport, because the blend films have been exposed to ambient air for a significant amount of time. The selective wet-etching process to the polymer and blend films has a minor effect on the mobility change. The devices based on P3HT, P3HT:PCBM, and P3HT:PCBM with DIO have the hole mobilities of 0.0099 (±0.0002) cm^2^ V^−1^ s^−1^, 0.0142 (±0.0040) cm^2^ V^−1^ s^−1^, and 0.0661 (±0.0024) cm^2^ V^−1^ s^−1^, respectively, after etching. With BA dipping, the threshold voltages of the corresponding devices shifted from −12.5 (±2.1) V to −28.2 (±4.7) V, from −23.3 (±2.4) V to −23.1 (±3.4) V, and from −0.7 (±6.7) V to −7.3 (±2.7) V, respectively. We note that we have not observed any evidence of remaining DIO in our sample after film processing (Figure S1). In addition, we have handled the devices under conventional laboratory environment and have not observed any evidence of photo-oxidation or degradation of the semiconductor caused by the DIO (Figures S2 and S3), although there may be a concern of radical formation caused by DIO under intense light (i.e., solar cell testing conditions) [48]. Under our experimental conditions, the device characteristics are reproducible and are not significantly affected by the extrinsic factors (Figures S4 and S5). 

**Figure 6:**
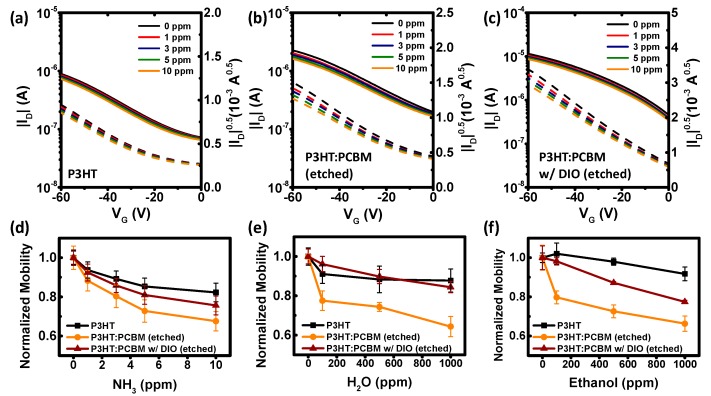
Sensor characteristics of OTFTs based on morphology-controlled P3HT films. (**a**–**c**) Transfer curves of sensor devices in vaporized ammonium hydroxide: (**a**) the pristine P3HT film, (**b**) the blend film after selective etching, and (**c**) the DIO-added blend film after etching. (**d**–**f**) Normalized hole mobility of various devices as a function of the analyte concentration: (**d**) NH_3_, (**e**) H_2_O, and (**f**) ethanol. Device testing conditions: *W*/*L* = 20 and *V*_D_ = −60 V.

We tested the surface- and morphology-controlled OTFT devices for gas-phase sensors, because the sensitivity of chemical sensors could be enhanced by the control of surface and morphology of thin polymer films (Figure 6). Because our blending and etching processes produce thin films with a rough surface with a large surface area and crystalline structures in a nanometer scale, analytes can easily make a contact with and penetrate into the electrically-active channel of the polymer layer. The analytes of interest in this work are ammonia, water, and ethanol, as these materials are commonly observed in living, industrial, and agricultural environments. Representative current–voltage curves of OTFT sensors upon ammonia exposure are shown in Figure 6a–c. Regardless of the film structures, the on-state drain current of the P3HT devices decreased as the ammonia concentration increased from 0 ppm to 10 ppm, as expected from the basic nature of the analyte [11,14]. Interestingly, the morphological changes amplified the amount of current decrease. As the charge-carrier mobility of transistor devices has been a convenient parameter of responses, we plotted the normalized field-effect mobility of the different sensor devices in ammonia environment to quantitatively compare the trends and sensitivity (Figure 6d). The mobility represents how the charge carriers transport in the channel and, unlike the electrical current and voltage, is fairly independent of the device and testing conditions [7,9]. The mobility of the pristine P3HT-based sensors dropped to 82.3% of the original value when exposed to 10 ppm of ammonia. The P3HT sensors after PCBM blending and etching are more sensitive. The relative mobilities of P3HT:PCBM devices after etching became 67.6% without DIO and 75.6% with DIO in a 10-ppm condition, compared to the devices in an inert nitrogen condition. If we quantify the sensitivity by using the degree of mobility reduction, the values for the pristine P3HT film, the PCBM-removed film, and the film processed with DIO and BA become 17.7%, 32.4%, and 24.4%, respectively, at 10 ppm of ammonia. We believe that, because the combinations of PCBM and DIO promotes the crystalline features of P3HT in thin films, the chance for analyte penetration in the more crystalline film decreases, resulting in a rather lower sensitivity. The morphology-controlled sensors can detect even 1 ppm of ammonia with a mobility reduction of 11.8%.

The decrease in the mobility after blending and etching was also observed in the case of water vapor or ethanol as the analyte (Figure 6e,f). With 1000 ppm of H_2_O, the hole mobility of the pristine P3HT film maintained the 87.8% level relative to the original value in nitrogen, marking the reduction of only 12.2%. When the P3HT:PCBM film was etched, the sensitivity to H_2_O increased as the mobility became 64.3% at 1000 ppm. At 1000 ppm of ethanol, the mobility values were reduced to 91.7% without the blending/etching processes, and to 66.3% after blending and etching. Although the degree of reduction is much smaller than ammonia because of the difference in selectivity of P3HT against the analytes, a similar tendency is observed: The etched P3HT:PCBM films show a better sensitivity compared to the pristine P3HT films. We note that the devices would respond to other analytes with different degrees depending on the nature of analytes and interactions. Therefore, complementary approaches using an array of semiconductors are essential for practical applications. Nevertheless, the results in this work demonstrate that the sensitivity of chemical sensors can be enhanced by modulating the nanostructures of the polymer films by a simple solution-based processing method.

## 4. Conclusions

We demonstrated that a sequential blending and selective etching processing can be a simple yet powerful method to control the surface and nanoscale morphology of thin polymer films. Molecular ordering within a P3HT domain is promoted in a blend with PCBM and DIO during film deposition. Such a nanostructural modulation enhances the charge transport properties of thin polymer films. By etching a small-molecular component from the blends of P3HT:PCBM, a rough and structured surface of thin films can be easily created. The surface and nanoscale morphology obtained through our procedure turns out to be beneficial for a better sensitivity in chemical sensing applications. Because our solution-based strategy is scalable, we believe that high-performance chemical sensors can be realized, with a development of novel polymers having a better specificity and selectivity towards a certain target analyte.

## Supplementary Materials

The following are available online at https://www.mdpi.com/2073-4360/11/10/1682/s1, Figure S1: XPS data, Figure S2: Stability of UV/Vis spectra, Figure S3: Temporal peak changes of UV/Vis spectra, Figure S4: Multiple current-voltage curves of OTFTs, Figure S5: OTFT results in nitrogen and in air.
