# Supplementary material for: Selective Wet-Etching of Polymer/Fullerene Blend Films for Surface- and Nanoscale Morphology-Controlled Organic Transistors and Sensitivity-Enhanced Gas Sensors
Source: Polymers (Basel). 2019 Oct 15;11(10):1682. doi: 10.3390/polym11101682 (PMC6836219; doi:10.3390/polym11101682)
Supplement: Supplementary file 1 [file polymers-11-01682-s001.pdf]

Supporting Information

Selective Wet-Etching of Polymer/Fullerene Blend Films for Surface- and Nanoscale Morphology-Controlled Organic Transistors and Sensitivity-Enhanced Gas Sensors

Min Soo Park, Alem Araya Meresa, Chan-Min Kwon, and Felix Sunjoo Kim*

School of Chemical Engineering and Materials Science, Chung-Ang University, Seoul 06974, Korea

**Corresponding author email: fskim@cau.ac.kr*

Figure S1. XPS results on the thin polymer and blend films: (a) Survey scans. (b) High-resolution scans for Iodine (I3d). (c) High-resolution scans for copper (Cu2p). There is no evidence of a trace amount of iodide and copper components.

Figure S2. UV/Vis absorption of polymer and blend films under different processing and light-exposure conditions.

Figure S3. Peak intensity changes of UV/Vis absorption of polymer and blend films under different processing and light-exposure conditions.

Figure S4. (a) Device-to-device variations of 10 OTFTs based on P3HT. (b) Ten consecutive scans of a single P3HT-based OTFTs. Device testing conditions: W/L=10 and V_D= –100 V.

Figure S5. Transfer curves of OTFTs based on a PCBM-washed films from a P3HT:PCBM blend with DIO, tested in nitrogen-filled chamber and in ambient air. The field-effect mobility values in nitrogen and in air are 0.0097 cm^2/Vs and 0.0093 cm^2/Vs, respectively. The device was also exposed to ambient laboratory conditions for multiple times for chamber-to-chamber transfer. Device testing conditions: W/L=20 and V_D= –60 V.

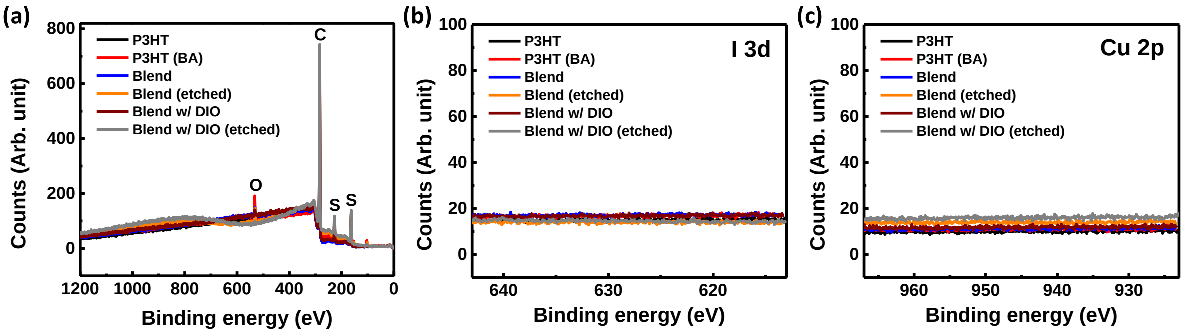

Figure S1. XPS results on the thin polymer and blend films: (a) Survey scans. (b) High-resolution scans for Iodine (I3d). (c) High-resolution scans for copper (Cu2p). There is no evidence of a trace amount of iodide and copper components.

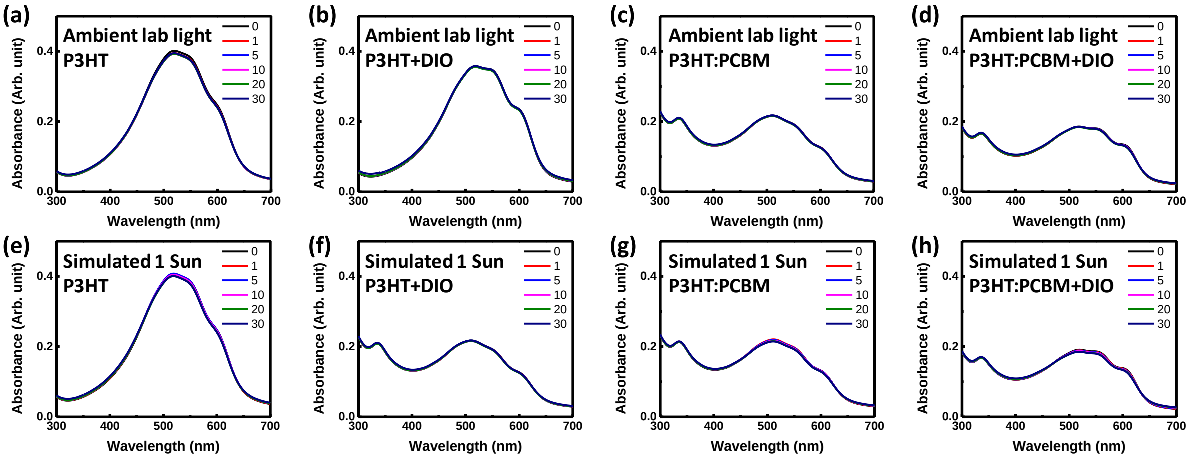

Figure S2. UV/Vis absorption of polymer and blend films under different processing and light-exposure conditions.

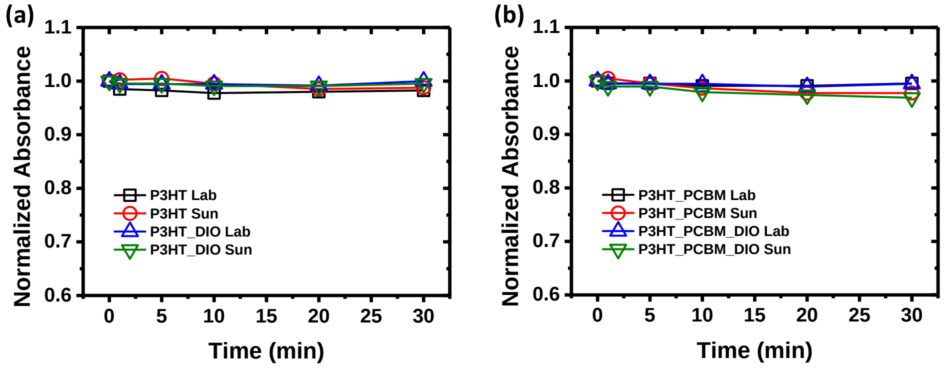

Figure S3. Peak intensity changes of UV/Vis absorption of polymer and blend films under different processing and light-exposure conditions.

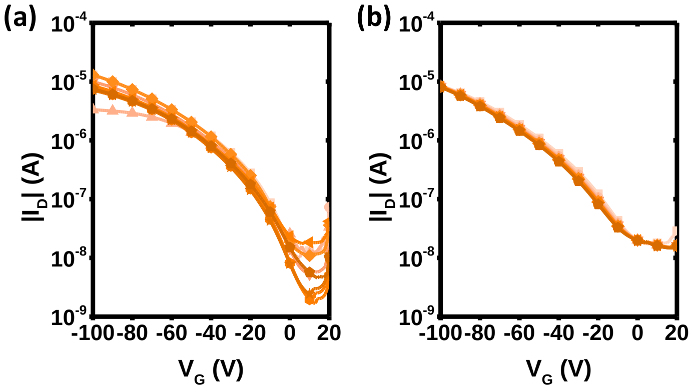

Figure S4. (a) Device-to-device variations of 10 OTFTs based on P3HT. (b) Ten consecutive scans of a single P3HT-based OTFTs. Device testing conditions: W/L=10 and V_D= –100 V.

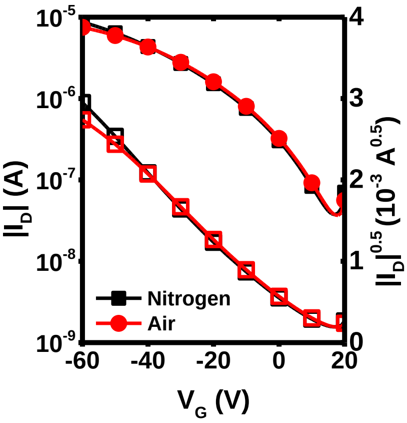

Figure S5. Transfer curves of OTFTs based on a PCBM-washed films from a P3HT:PCBM blend with DIO, tested in nitrogen-filled chamber and in ambient air. The field-effect mobility values in nitrogen and in air are 0.0097 cm^2/Vs and 0.0093 cm^2/Vs, respectively. The device was also exposed to ambient laboratory conditions for multiple times for chamber-to-chamber transfer. Device testing conditions: W/L=20 and V_D= –60 V.
